# Relationship between academic success, distance education learning environments, and its related factors among medical sciences students: a cross-sectional study

**DOI:** 10.1186/s12909-023-04856-3

**Published:** 2023-11-09

**Authors:** Saeed Ghasempour, Maede Esmaeeli, Ali Abbasi, Ali Hosseinzadeh, Hossein Ebrahimi

**Affiliations:** 1 Student Research Committee, School of Nursing and Midwifery, Shahroud University of Medical Sciences, Shahroud, Iran; 2 Department of Nursing, School of Nursing and Midwifery, Shahroud University of Medical Sciences, Shahroud, Iran; 3 Department of Epidemiology, School of Public Health, Shahroud University of Medical Sciences, Shahroud, Iran; 4 Center for Health Related Social and Behavioral Sciences Research, Shahroud University of Medical Sciences, Shahroud, Iran

**Keywords:** Academic success, Learning Environment, Distance Education, Students

## Abstract

**Background:**

Academic success is among the most important criteria for determining students’ competence. Hence, one of the concerns of education system researchers has always been investigating its associated factors. Therefore, this study aimed to determine the relationship between academic success, distance education learning environments, and its related factors among Shahroud University of Medical Sciences students.

**Methods:**

This cross-sectional study was conducted on 208 medical sciences students who completed at least two online and two in-person academic semesters. Participants were selected through the convenience sampling method and filled out three questionnaires, including the demographic information form, the Academic Success Inventory for College Students, and the Distance Education Learning Environments Survey. Finally, the data were analyzed using descriptive statistics and inferential tests (t-test, ANOVA, Pearson’s correlation coefficient, and multiple linear regression).

**Results:**

In this study, students reported moderate levels of academic success (107.81 ± 10.72). Moreover, they assessed their distance education learning environment as the positive points were more than the negative points (74.10 ± 14.89). Distance education learning environment (*β =* 0.233 and *P <* 0.001) and field satisfaction (*β =* 9.797 and *P =* 0.001) were also mentioned as factors related to students’ academic success.

**Conclusion:**

According to the present results, it is suggested to improve the learning environment of distance education and increase students’ satisfaction to enhance their academic outcomes such as academic success.

**Supplementary Information:**

The online version contains supplementary material available at 10.1186/s12909-023-04856-3.

## Introduction

Students’ academic success in the university is one of the essential factors in their long-term career and social growth in life [1]. Additionally, it is one of the most critical concerns in any educational system [2, 3]. Defining academic success is challenging [4]. Some papers define it as gaining knowledge and skills in a specific field and completing an academic course [5]. Some consider students’ grade point average (GPA) a measure of their success [6], and others believe that the university has duties beyond educating theoretical knowledge and should be able to create special abilities in students, especially in terms of spiritual-psychological, social, and scientific abilities [7, 8]. However, York et al. (2015) defined academic success as acquiring the necessary knowledge and skills with the specified achievements to complete the courses, which includes six components: “Academic achievement, satisfaction, acquisition of skills and competencies, persistence, attainment of learning objectives, and career success” [5]. This complex concept includes objective and subjective dimensions. The objective dimension is the GPA and the student’s opinion about academic status. The subjective dimension includes academic satisfaction, persistence, and the perception of success in education [9].

Based on previous studies, one of the factors affecting students’ academic success is the learning environment [10–13]. The learning environment is defined as: “…social interactions, organizational cultures and structures, and physical and virtual spaces that surround and shape participants’ experiences, perceptions, and learning.” [14] In this regard, one of these learning environments is the learning environment of students’ distance education. During the COVID-19 pandemic, medical sciences universities directed students out of the hospital and the university toward distance education to comply with health protocols. They suspended most of their face-to-face activities and resorted to remote activities using online communication [15–17]. This sudden transition has been associated with challenges for universities and students [16, 18, 19]. Among these challenges are the deprivation of face-to-face training and meaningful clinical experiences in medical sciences [18], disruption of students’ daily schedules, nutritional disorders [20], depression, anxiety, and stress [21–23]. In Nepal, Kalauni et al. (2023) showed that 50.5%, 52.5%, and 44.6% of undergraduate health sciences students experienced symptoms of depression, anxiety, and stress during the COVID-19 pandemic, respectively [24]. Additionally, Yuan et al. (2021) reported the prevalence of anxiety and depressive symptoms in Chinese international medical students during this pandemic as 28.5% and 31.6%, respectively [25].

Examining the influential factors and determining the contribution of each one to academic progress helps determine strategies to identify the effective factors in academic success [26]. Various factors, such as intellect [27], progress motivation [28], mental health [29], academic self-efficacy, academic skill, academic enthusiasm [30], social support [31], academic procrastination [32], and career path adaptability [33], affect students’ academic success. According to Bayat et al. (2019), there was no significant relationship between the age and level of education of students and their academic success score, while the effect of marital status and place of residence on students’ academic success was significant [34]. Ghadirzadeh et al. (2017) demonstrated that among all demographic characteristics, only ethnicity and housing status (dormitory and nondormitory) are effective factors in the academic success of students, while age, gender, field, and academic semester did not show a significant relationship with students’ academic success [35]. However, contradictory results raise questions about the individual or social factors related to student academic success [36].

Assessing the factors associated with academic success has always been one of the concerns of researchers in the education system [37–39]. In addition, the COVID-19 pandemic caused significant disruptions and challenges to the students’ learning environment, and many were driven entirely to distance education. Hence, barriers to academic success that already exist in face-to-face and traditional classrooms may be compounded by this environment and exacerbated by pandemic-related stressors [40], which many believe harmed their academic success [41]. Until now, studies have yet to determine the relationship between academic success and the learning environment of students’ distance education during this pandemic. Therefore, this study aimed to determine the relationship between academic success, distance education learning environments, and its related factors among Shahroud University of Medical Sciences students.

## Materials and methods

### Research questions and hypotheses

This study primarily aimed to determine the level of academic success and its relationship with the distance education learning environment. As a secondary aim, this study also tries to identify the possible relationship between academic success and demographic variables such as age, gender, marital status, academic semester, field of study, level of satisfaction with the field, income adequacy, residence status, and student’s GPA. Therefore, in addition to determining the student’s academic success rate, the following hypotheses will also be tested:

Q_1_: What is the academic success rate of Shahroud University of Medical Sciences students?

H_1_: There is an association between academic success and distance education learning environment.

H_2_: There is an association between academic success and age.

H_3_: There is an association between academic success and gender.

H_4_: There is an association between academic success and marital status.

H_5_: There is an association between academic success and the academic semester.

H_6_: There is an association between academic success and field of study.

H_7_: There is an association between academic success and field satisfaction.

H_8_: There is an association between academic success and income adequacy.

H_9_: There is an association between academic success and residence status.

H_10_: There is an association between academic success and GPA.

**Fig. 1:**
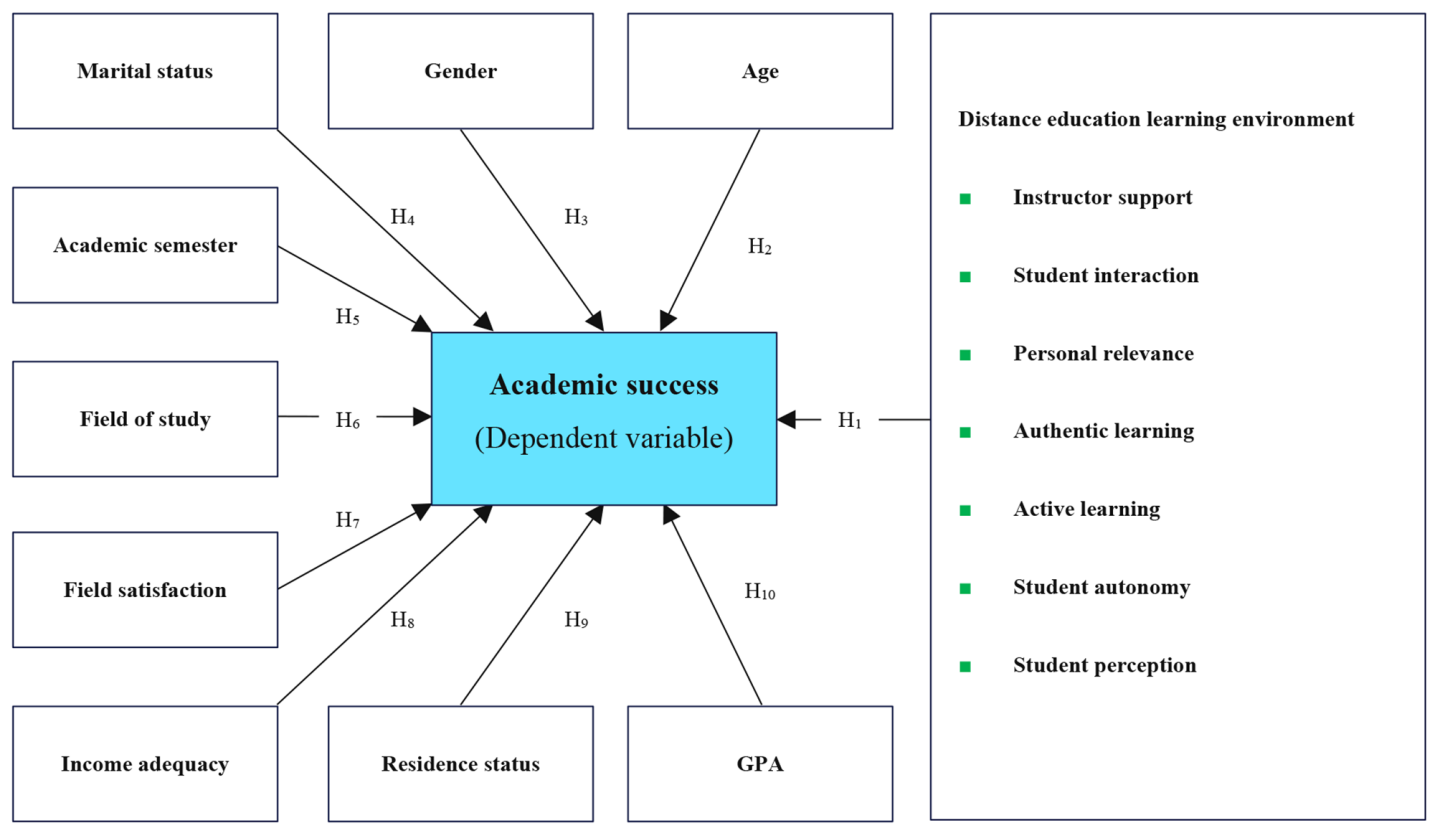
Conceptual framework of the study

The study’s conceptual framework to better understand the research questions and hypotheses is given in Fig. 1.

### Study design and participants

This cross-sectional study was performed in the first half of the academic year 2022–2023 on 208 students of Shahroud University of Medical Sciences. Students who completed at least two academic semesters online and two in-person were included using the convenience sampling method. The sample size was 208, calculated based on the study of Bayat et al. (2019) and considering the standard deviation of 10.63, the accuracy of 1.5 at the 95% confidence level [34], and the 8% dropout of samples.

$$n\, = \,\frac{{{\rm{ }}z_{1\, - \,\frac{{{\rm{ }}\alpha {\rm{ }}}}{{{\rm{ }}2{\rm{ }}}}}^2\,{\sigma ^2}}}{{{\rm{ }}{d^{{\rm{ }}2}}{\rm{ }}}}\, = \,\frac{{{{(1.96)}^2}\,{{(10.63)}^2}}}{{{{(1.5)}^2}}}\, = \,193$$

### Measurements

The data collection tools in this study were three questionnaires:

#### Demographic information form

In this form, information about age, gender, marital status, academic semester, field of study, level of satisfaction with the field, income adequacy, and student’s residence were asked. Notably, the total GPA (the minimum and maximum scores were 0 and 20, respectively) of all participating students was also obtained from the Informatics Unit of the University.

#### Academic success inventory for college students (ASICS)

Students’ academic success was measured using the ASICS instrument. This questionnaire consists of 39 items and ten components, including general academic skills (items 1–7), instructor’s effectiveness (items 8–11), career decision (items 12–14), external motivation for the future (items 15–18), trust (items 19–23), personal adjustment (item 24), self-regulation (items 25–27), socializing (items 28–31), internal motivation or interest (items 32–36) and lack of anxiety (items 37–39), designed by Welles, which is graded on a four-point scale (Completely agree = 4, Agree = 3, Disagree = 2 and Completely disagree = 1). Reverse items of this questionnaire were 9, 10, 11, 19, 24, 26, 27, 28, 29, 30, 31, 36, 37, 38, and 39 (“Completely agree” with a score of 1 and “Completely disagree” with a score of 4). To calculate the score of each component or subscale, the score of each item related to that subscale must be added together. Additionally, to calculate the overall score of the questionnaire, the scores of all questionnaire items must be added together. The score range of this questionnaire will be between 39 and 156. The higher the score obtained from this questionnaire, the higher the academic success. Welles et al. (2010) confirmed this questionnaire’s face, content, and construct validity and reported its reliability using Cronbach’s alpha method, 0.93 [7]. In the study by Adib-Hajbaghery et al. (2015), first, this questionnaire was translated into Persian using the process of translation and retranslation. Then, its face validity was confirmed by students, and several experts confirmed its content validity. In addition, its total reliability coefficient was calculated using Cronbach’s alpha method as 0.76 [42]. In the present study, the reliability of the Persian version of this questionnaire was 0.84 by Cronbach’s alpha method.

#### Distance education learning environments survey (DELES)

The DELES questionnaire was used to evaluate students’ distance education learning environment. This tool has 30 questions, with seven principal components, including instructor support (questions 1 to 7), student interaction (questions 8 to 11), personal relevance (questions 12 to 14), authentic learning (questions 15 to 17), active learning (questions 18 to 21), student autonomy (questions 22 to 24) and student perception (questions 25 to 30). The scoring scale of the DELES questionnaire is based on a five-point Likert scale (Never = 0, Rarely = 1, Sometimes = 2, Often = 3, Always = 4), and the range of scores obtained from this questionnaire will be between 0 and 120 points. Comprehension is feeble if the score is between 0 and 30. If the obtained score is between 31 and 60, the concept of maximum score among the set of problems is received. If the score is between 61 and 90, the positive points are more than the negative points, and if the score is between 91 and 120, it indicates an excellent condition [43]. The reliability of this questionnaire was obtained by Walker et al. (2005) using Cronbach’s alpha between 0.75 and 0.94. Its construct validity was also investigated using the factor analysis method (principal component analysis with varimax rotation and Kaiser normalization) [44]. This questionnaire was first translated into Persian and retranslated by Kuhpayehzadeh et al. (2017). Then, its face validity was confirmed by students, and several experts confirmed its content validity. Cronbach’s alpha of the Persian version of this questionnaire was reported as 0.93 [43]. The reliability of the Persian version of this questionnaire in the present study was also 0.89 by Cronbach’s alpha method.

### Ethical considerations

First, the necessary permissions were obtained from the Vice President of Research and Technology and the Research Ethics Council of Shahroud University of Medical Sciences (Ethics code: IR.SHMU.REC.1401.051). Additionally, the necessary cooperation was made with the officials of all four nursing and midwifery, medicine, allied medical sciences, and public health faculties and the directors of each academic field. Then, the study objectives and the link to the relevant questionnaires were placed in the students’ groups and study channels, and they were asked to complete the questionnaires in their free time.

### Statistical analysis

The data were analyzed using descriptive statistics (frequency, percentage, mean, and standard deviation) and inferential tests (t-test, analysis of variance, Pearson correlation coefficient, and multiple linear regression) in SPSS software version 16. The significance level of the tests was considered 0.05.

## Results

**Fig. 2:**
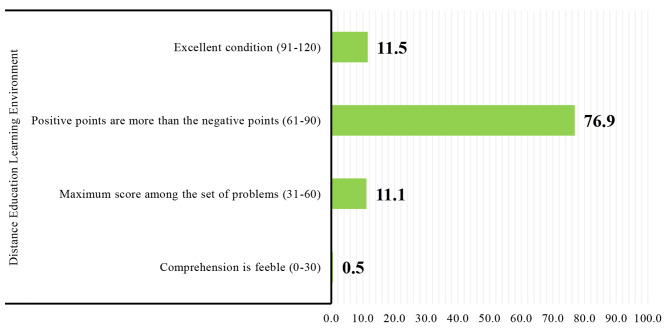
The level of students’ distance education learning environment

Two hundred eight students from Shahroud University of Medical Sciences participated in the present study, of which most students (61.5%) were women. The mean and standard deviation of the participating students’ age, GPA, and academic semester were 21.88 ± 2.43, 16.31 ± 1.26, and 5.80 ± 1.94, respectively. Additionally, the mean score of students’ academic success was 107.81 ± 10.72. Demographic characteristics and mean scores of students’ academic success are shown separately in Table 1. Students evaluated the mean score of their distance education learning environment as 74.10 ± 14.89. The mean scores of academic success and distance education learning environment of students by their components are given in Additional file 1. The different levels of students’ distance education learning environment are also shown in Fig. 2.

**Table 1:** The mean scores of academic success based on demographic distribution

Variable		N	%	Academic success		
				Mean	SD	P
Gender	Male	80	38.5	107.70	9.25	0.909^*^
	Female	128	61.5	107.88	11.57	
Marital status	Married	10	4.8	110.70	11.61	0.383^*^
	Single	198	95.2	107.66	10.68	
GPA	12–14	10	4.8	105.30	9.66	0.150^**^
	14–17	115	55.3	106.46	11.34	
	17<	61	29.3	109.64	10.04	
Academic year	Second year	63	30.3	109.40	10.69	0.208^**^
	Third year	126	60.6	107.64	10.25	
	Fourth-year and above	16	7.7	104.25	13.97	
School	Nursing and Midwifery	85	40.9	107.49	10.29	0.011^**^
	Medicine	48	23.1	104.25	10.48	
	Allied Medical Sciences	59	28.4	109.78	11.71	
	Public Health	16	7.7	112.88	5.80	
Residence status	Dormitory	126	60.6	107.36	10.83	0.370^**^
	Being with family	66	31.7	107.80	10.57	
	Rental house	16	7.7	111.38	10.38	
Field Satisfaction	Dissatisfied	15	7.2	102.20	13.82	< 0.001^**^
	Unsure	79	38.0	104.29	9.09	
	Satisfied	114	54.8	110.98	10.33	
Income adequacy	Below average	63	30.3	106.81	10.90	0.592^**^
	Average	141	67.8	108.16	10.63	
	Above average	4	1.9	111.00	12.54	

** N**: Frequency; **%**: Percent; **SD**: Standard Deviation; **P**: P-value; **GPA**: Grade Point Average; *: Independent t-test; **: One-way ANOVA

Based on the results, the mean score of students’ academic success according to gender, marital status, GPA, academic year, place of residence, and income adequacy did not have a statistically significant difference. The results of the one-way analysis of variance showed that the mean score of students’ academic success in different faculties was significantly different (*P =* 0.011). Tukey’s post hoc test was used to track the difference between groups. Based on this, medical students had lower academic success mean scores than public health (*P =* 0.025) and allied medical sciences (*P =* 0.037) students. In addition, the mean score of academic success of students according to their satisfaction with their field of study also had a statistically significant difference (*P <* 0.001), so that students with high satisfaction compared to students with moderate (*P <* 0.001) and low satisfaction (*P =* 0.005) reached a higher level of mean scores in academic success.

Pearson’s correlation coefficient results found a positive and significant correlation between academic success, distance education learning environment, and all its principal components except student perception (*P <* 0.05). A negative and significant correlation was also between academic success and student perception (*P >* 0.01 and *r=*-0.189). Pearson correlation coefficient results are shown in Table 2.

**Table 2:** Correlation matrix of academic success, distance education learning environment, and its seven principal components

Variables	Academic success	Distance education learning environment	Instructor support	Student interaction	Personal relevance	Authentic learning	Active learning	Student autonomy	Student perception
Academic success	1	0.306^**^	0.388^**^	0.167^*^	0.455^**^	0.357^**^	0.470^**^	0.308^**^	-0.189^**^
Distance education learning environment		1	0.679^**^	0.588^**^	0.594^**^	0.706^**^	0.656^**^	0.555^**^	0.566^**^
Instructor support			1	0.374^**^	0.409^**^	0.427^**^	0.407^**^	0.346^**^	0.090
Student interaction				1	0.493^**^	0.417^**^	0.323^**^	0.293^**^	0.068
Personal relevance					1	0.685^**^	0.557^**^	0.415^**^	-0.085
Authentic learning						1	0.571^**^	0.418^**^	0.156^*^
Active learning							1	0.709^**^	0.055
Student autonomy								1	0.019
Student perception									1

**: Correlation is significant at the **0.01** level (2-tailed)

*: Correlation is significant at the **0.05** level (2-tailed)

According to the results of the multiple linear regression model using the Enter method, 18.17% of the variance of academic success was explained by the variables inside the model. This model showed that for each unit of increase in distance education learning environment, students’ mean academic success score increases by 0.233 units (*P <* 0.001). In addition, students who were more satisfied with their field of study had 9.797 units of higher academic success than students with less satisfaction (*P =* 0.001). The role of other independent variables on students’ academic success is given in Table 3.

**Table 3:** The role of independent variables on academic success based on the multiple regression model

**Independent variable**	**Dependent variable**	**Academic success**			
		β	SE	t	P
GPA	12–14 (Reference)				
	14–17	0.73	3.444	0.21	0.832
	17<	2.268	3.663	0.62	0.537
Academic year	Second year (Reference)				
	Third year	0.915	1.967	0.47	0.642
	Fourth-year and above	-0.247	3.632	-0.07	0.946
Gender	Male (Reference)				
	Female	-1.23	1.614	-0.76	0.447
Marital status	Married (Reference)				
	Single	1.544	3.82	0.4	0.687
Age		0.013	0.531	0.02	0.98
School	Nursing and Midwifery (Reference)				
	Medicine	-1.321	2.268	-0.58	0.561
	Allied Medical Sciences	2.155	1.976	1.09	0.277
	Public Health	5.61	3.394	1.65	0.1
Residence status	Dormitory (Reference)				
	Being with family	-0.021	1.701	-0.01	0.99
	Rental house	4.063	2.799	1.45	0.148
Field satisfaction	Dissatisfied (Reference)				
	Unsure	4.257	3.145	1.35	0.178
	Satisfied	9.797	2.971	3.3	0.001
Income adequacy	Below average (Reference)				
	Average	0.801	1.682	0.48	0.635
	Above average	4.078	6.046	0.67	0.501
Distance education learning environment		0.233	0.054	4.33	< 0.001
(Constant)		78.596	17.309	4.54	< 0.001

**SE**: Standard Error; **P**: P-value; **GPA**: Grade Point Average

## Discussion

The present study was performed to determine the relationship between academic success, distance education learning environments, and its related factors among medical sciences students. Students in this study obtained a mean academic success score of 107.81 ± 10.72. In the study of Bayat et al. (2019) on Tehran University of Medical Sciences students, it was 108.87 ± 10.63, consistent with the present study’s results [34]. Meanwhile, in the study of Ghadirzadeh et al. (2017), nursing and midwifery students of Kashan University of Medical Sciences obtained a mean academic success score of 131.14 ± 41.10 [35]. It is worth noting that this study was performed before the COVID-19 pandemic and in face-to-face training. Hence, distance education in the wake of this pandemic and its adverse effect on students’ academic success may be among the possible reasons for this difference.

In this study, the majority of students listed the strengths of their distance education learning environment more than their weaknesses. In this regard, Lin et al.‘s (2021) study showed that contrary to expectations, after the onset of the COVID-19 pandemic, students had a more positive perception of their learning environment than before the beginning of this pandemic [45]. In Khalil et al.‘s (2020) study, most students positively perceived online learning during this sudden transition following the COVID-19 pandemic [46]. These findings indicate this educational method’s significant and promising potential to deal with critical and unpredictable conditions such as COVID-19.

A positive and significant correlation was also found between academic success and students’ distance education learning environment. In this regard, Omoniyi-Esan et al. (2022) found the learning environment to be one of the factors affecting students’ academic success. Hence, students’ experiences of their learning environment had a positive and significant relationship with their academic success, achievement, and satisfaction [10]. Additionally, Al-Qahtani (2015) mentioned the students’ approaches to studying and their educational environment as important factors that affect their learning and academic achievement [47]. Various studies also showed that the perceived educational environment affects academic outcomes such as academic achievement, well-being, social-emotional adjustment, and self-esteem of students [48–50]. Hence, an efficient learning platform helps students cope with distance education’s obstacles and challenges more easily and take advantage of this environment to improve their academic status. Therefore, educational managers and relevant officials can play a crucial role in promoting students’ academic success by creating this platform.

Contrary to what was expected, student perception, one of the components of the distance education learning environment, negatively correlated with academic success. However, in Ahmed et al.‘s (2018) study, which evaluated students’ perception of the learning environment based on the DREEM model and its relationship with their study year and academic performance, students with higher academic achievement had a more positive attitude towards their education, while students with lower academic achievement showed a more negative perception of education [51]. On the other hand, in the study of Al-Ansari et al. (2015), which was conducted to evaluate dental students’ perception of the educational environment based on the mentioned model and its relationship with their academic performance, there was no significant relationship between students’ perception of this environment and their academic performance, which was measured using GPA [52]. The possible reasons for this discrepancy are the difference in the face-to-face or distance nature of education and the tools for measuring students’ perception of the learning environment.

Other reasons for this contradiction can be found in other studies. In this regard, Jafari Asl et al. (2015) found that students’ expectations of the quality of educational services exceeded their perceptions, so the quality of these services was considered inappropriate from the students’ point of view [53]. In addition, in the study of Tofighi et al. (2011), which was performed to determine the gap in the quality of educational services based on the ServQual model at the Tehran University of Medical Sciences, students’ expectations of the quality of educational services were not met. There was a negative gap between the perceptions and expectations of students in all dimensions [54]. There may be a gap between students’ perceptions and expectations in this study, especially in students who experience higher levels of academic success. Therefore, the learning environment of distance education did not meet the expectations of these students regarding the quality of educational services provided, and they showed a more negative perception of this environment.

In the present study, there was no significant relationship between place of residence and students’ academic success. In this regard, SadeghiMovahed et al. (2013) considered the place of residence as one of the factors related to students’ academic success, so native students reported higher levels of academic success [55]. However, Adib-Hajbaghery et al. (2015) indicated that nonnative students living in the dormitory had higher academic success [42]. In justifying these contradictory results, distance education and online teaching on platforms such as Skyroom and Adobe Connect and the lack of location restrictions on these platforms should be mentioned. Hence, it is expected that during the COVID-19 pandemic and distance education, students with and without residence in a dormitory will experience similar levels of academic success.

The results of the present study demonstrated that the increase in satisfaction with the students’ field of study leads to higher academic success. Saravand et al. (2013) showed that successful students were more satisfied with their field of study than unsuccessful students [56]. Moreover, Kim et al. (2015) stated that satisfaction with the field of study is one of the factors affecting students’ academic success [57]. Younas et al.’s (2022) study conducted during the COVID-19 pandemic showed a positive and significant relationship between satisfaction with e-learning and students’ academic achievement [58]. Kim et al. (2022) also found satisfaction with online classes during this pandemic to be one of the factors influencing undergraduate nursing students’ academic achievement [59]. This satisfaction seems to increase students’ motivation, effort, and perseverance, which are essential in improving their academic performance. Therefore, it is expected that students who are more satisfied with their field and course of study will experience higher academic success.

In the present study, medical students obtained the lowest mean academic success score compared to other students. Intensive and complex educational curricula, challenging internship courses, frequent exams, and fear of failure have made the field of medicine one of the most stressful fields of study [60, 61]. Todres et al. (2012) listed the compactness of course units and the lack of sufficient time to study courses as obstacles to the academic success of medical students [62]. In addition, during the COVID-19 pandemic, these students were deprived of many important clinical experiences, and a large part of their educational curriculum was presented virtually [18], which many believe has hurt their academic performance [41]. Therefore, students in this popular field are expected to have lower academic success than others.

### Research limitations and recommendations

It should be noted that the present study was only performed on students of Shahroud University of Medical Sciences, making it difficult to generalize the results to nonmedical sciences universities in the country. Therefore, similar studies should be performed with a longitudinal design and a larger sample size in the future. It is also recommended that future studies investigate the impact of different learning methods on students’ academic success.

## Conclusion

Students in this study experienced moderate levels of academic success. Additionally, they listed the strengths of their distance education learning environment more than its weaknesses. There was a positive and significant correlation between academic success and this environment. Satisfaction with the field of study was also mentioned as another factor related to students’ academic success.

These findings suggest that improving students’ distance education learning environment plays an important role in enhancing their academic success and dealing with possible critical and unpredictable conditions such as COVID-19. Therefore, educational managers and relevant officials can play a crucial role in improving the learning environment and academic outcomes, such as academic success, by holding group discussion meetings to understand educational problems, increase student satisfaction, and create constructive interaction to create an ideal and satisfactory learning environment.

### Electronic supplementary material

Below is the link to the electronic supplementary material.

Supplementary Material 1

## Data Availability

The datasets used and/or analyzed during the current study are available from the corresponding author upon reasonable request.

## Abbreviations

ANOVA: Analysis of variance
ASICS: Academic Success Inventory for College Students
DELES: Distance Education Learning Environments Survey
DREEM: Dundee Ready Education Environment Measure
GPA: Grade point average
ServQual: Service Quality
