# Supplementary material for: Relationship between academic success, distance education learning environments, and its related factors among medical sciences students: a cross-sectional study
Source: BMC Med Educ. 2023 Nov 9;23:847. doi: 10.1186/s12909-023-04856-3 (PMC10633937; doi:10.1186/s12909-023-04856-3)
Supplement: Supplementary file 1 — Supplementary Material 1 [file 12909_2023_4856_MOESM1_ESM.pdf]

The mean score of academic success and distance education learning environment by their components

Variables		Min	Max	Mean	SD
**Academic success**	**Total**	39	134	107.81	10.72
	General academic skills	7	28	19.71	3.31
	Instructor's effectiveness	4	16	9.34	1.91
	Career decision	3	12	8.62	1.82
	External motivation for the future	4	16	12.73	2.18
	Trust	5	20	15.27	1.93
	Personal adjustment	1	4	2.41	0.72
	Self-regulation	3	12	7.19	1.80
	Socializing	4	16	10.33	1.71
	Internal motivation or interest	5	20	14.01	2.49
	Lack of anxiety	3	12	8.20	2.23
**Distance education learning environment**	**Total**	0	120	74.10	14.89
	Instructor support	0	28	17.61	4.71
	Student interaction	0	16	10.35	3.03
	Personal relevance	0	12	7.56	2.32
	Authentic learning	0	12	6.86	2.24
	Active learning	0	16	11.54	2.53
	Student autonomy	0	12	9.39	1.91
	Student perception	0	24	10.79	7.47

Min: Minimum; **Max**: Maximum; **SD**: Standard Deviation
